# Twenty-six-year trends in U.S. mortality among older adults with coexisting chronic kidney disease and atrial fibrillation/flutter

**DOI:** 10.1097/MS9.0000000000004960

**Published:** 2026-04-27

**Authors:** Sumet Kumar, Rakhshan Zulfiqar, Shiza Khan, Waqar Malik, Ayesha Rehman, Fnu Arshia, Muhammad Junaid Imran, Alisha Nouman, Hiba Naz, Laksh Kumar, Harmain Muhammad Asif, Tanzeela Khan, Adarsh Raja, Aayush Chaulagain

**Affiliations:** a Department of Medicine, Shaheed Mohtarma Benazir Bhutto Medical College Lyari, Karachi, Pakistan; b Department of Medicine, Karachi Medical and Dental College, Karachi, Pakistan; c Department of Medicine, Liaquat University of Medical and Health Sciences, Jamshoro, Pakistan; d Department of Medicine, Patan Academy of Health Sciences, Lalitpur, Nepal

**Keywords:** atrial fibrillation, chronic kidney disease, epidemiology, mortality trends, older adults

## Abstract

**Background:**

Chronic kidney disease (CKD) and atrial fibrillation/flutter (AF/AFL) frequently coexist in older adults, sharing common risk factors such as hypertension, diabetes, and coronary artery disease. However, long-term national mortality trends among patients with concurrent CKD and AF/AFL remain poorly defined. The primary research question of this study is to examine how the combined presence of CKD and AF/AFL has influenced mortality rates in older adults in the U.S. over the past 26 years.

**Methods:**

We analyzed mortality data from the CDC Wide-Ranging Online Data for Epidemiologic Research database for U.S. adults aged ≥65 years from 1999 to 2024. Deaths listing both CKD (ICD-10 N18.0–N18.9) and AF/AFL (I48) were identified. Age-adjusted mortality rates (AAMRs) per 100 000 population were calculated using the 2000 U.S. standard population and stratified by sex, race/ethnicity, census region, and urban–rural status. Temporal trends were assessed using Joinpoint regression to estimate annual percentage change (APC).

**Results:**

A total of 203 662 deaths were attributed to concurrent CKD and AF/AFL. The overall AAMR increased from 4.9 per 100 000 in 1999 to 30.0 per 100 000 in 2024. Mortality rose rapidly from 1999 to 2011 (APC: +12.3%, *P* = 0.0016), followed by a slower, non-significant increase from 2011 to 2024 (APC: +3.9%, *P* = 0.0767). Mortality was higher in men and in nonmetropolitan areas. Non-Hispanic White and Black populations showed the greatest increases, while Hispanic and Asian/Pacific Islander groups had the lowest rates. The Midwest and West exhibited the highest regional burdens, with notable state-level variation.

**Conclusions:**

Mortality linked to concurrent CKD and AF/AFL has risen sharply, with widening disparities by sex, race, and geography. Integrated, equitable care strategies are urgently needed.

## Introduction

An estimated 35.5 million Americans, or more than one in seven adults, suffer from chronic kidney disease (CKD)^[^1^]^. People 65 and older are more likely to have CKD (34%), compared to those 45–64 years old (12%) and 18–44 years old (6%), respectively^[^1^]^. In Americans with diabetes or high blood pressure, the two most prevalent causes of kidney disease, atrial fibrillation (AF), and CKD are closely related conditions that share risk factors like coronary artery disease, diabetes, and hypertension^[^2^]^.

HIGHLIGHTS

- A total of 203 662 deaths from CKD and atrial fibrillation/flutter in adults ≥65 years, 1999–2024.
- Highest rates in men, nonmetropolitan areas, and Midwest/West regions.
- Black and White populations showed the greatest increases; Hispanics lowest rates.

Heart rhythm abnormalities, such as ventricular arrhythmias, supraventricular tachycardias, atrial fibrillation/flutter (AF/AFL), and sudden cardiac death, are more common in patients with CKD^[^3^]^. The prevalence of AF, the most common sustained cardiac arrhythmia, is substantially higher among patients with CKD, with reported estimates ranging from 15% to 40% in dialysis-dependent individuals and from 16% to 21% in non-dialysis-dependent CKD patients^[^4^]^. People with AF are approximately twice as likely to experience stroke compared with those without AF, and both CKD and AF are independent risk factors for stroke^[^5^]^.

Long-term mortality patterns among older adults with coexisting AF/AFL and CKD remain insufficiently defined, despite the high and growing prevalence of this comorbidity. Existing literature has predominantly focused on cardiovascular or renal outcomes in isolation, offering limited insight into the combined mortality burden of AF/AFL and CKD at the national level. As population aging accelerates and multimorbidity becomes increasingly common, a clearer understanding of these trends is critical for guiding public health planning and resource allocation. However, comprehensive national analyses examining long-term mortality trends associated with the coexistence of AF/AFL and CKD, including variations by sex, race, and geographic region, are lacking. This study was designed to address this critical knowledge gap. This study adhered to the Strengthening the Reporting of Observational studies in Epidemiology (STROBE) guidelines for observational studies. No artificial intelligence (AI) tools were employed in the research design, data collection, analysis, or interpretation in compliance with the TITAN Guidelines 2025 for the transparent use of AI in scholarly communication; AI support was restricted to language improvement during manuscript preparation^[^6^]^.

## Methods

### Population and study setting

We conducted a population-based mortality analysis using data from the Centers for Disease Control and Prevention’s Wide-Ranging Online Data for Epidemiologic Research (CDC WONDER) database, which is derived from U.S. death certificate records. The study population included adults aged ≥65 years who died between 1999 and 2024, providing a 26-year observational period for assessment of long-term mortality trends. Decedents were included if their death certificates recorded both CKD and AF/AFL as either the underlying cause of death or as contributing (multiple) causes of death, as defined by the International Classification of Diseases, Tenth Revision (ICD-10). AF/AFL was identified using ICD-10 code I48, while CKD was identified using codes N18.0–N18.9, consistent with established CDC WONDER-based epidemiologic studies. Mortality data were obtained from the National Vital Statistics System, which captures all death certificates filed across the 50 U.S. states and the District of Columbia, thereby providing a complete census of U.S. mortality. Institutional review board approval was not required, as the analysis was conducted using de-identified, publicly available data.

### Data abstraction

The abstracted data included details about population counts, year, place of death, demographic characteristics, geographical segmentation, state-specific data, and distinction between urban and rural areas. The place where the death occurred encompassed medical facilities (inpatient, outpatient, or emergency room, death on arrival, and status unknown), nursing homes, or long-term facilities. “Demographics” refers to data on gender, age, race, and ethnicity. Race and ethnicity were designated as non-Hispanic (NH) White individuals, NH Black or African American individuals, Hispanic or Latino individuals, NH American Indian or Alaskan Native individuals, and NH Asian or Pacific Islander individuals. The data used in the analysis were sourced from death certificates, which have also been utilized in previous research employing the WONDER database^[^7^]^. The study utilized the National Center for Health Statistics Urban-Rural Classification Scheme to examine geographic variations in mortality patterns. Under this framework, urban regions were classified as large metropolitan areas (population ≥1 million) along with medium and small metropolitan areas (population 50 000–999 999). Rural areas (non-metropolitan area) comprised counties with populations below 50 000s, following the 2013 U.S. Census specifications^[^8^]^. This systematic classification enabled comparative analysis of disease burden across different community types. Furthermore, as per the US Census Bureau classification, the US was partitioned into four separate regions, namely the Northeast, Midwest, South, and West^[^9^]^.

### Statistical analysis

To analyze national trends in mortality related to AF/AFL and CKD among adults aged 65 and older from 1999 to 2024, we calculated both crude and age-adjusted mortality rates (AAMRs) per 100 000 population. These rates were stratified by year, sex, race/ethnicity, and urban-rural classification to identify potential disparities. Crude mortality rates were derived by dividing the annual number of AF/AFL-CKD-related deaths by the corresponding U.S. population for each year. AAMR were standardized to CKD with AF/AFL-related deaths in the U.S. population in 2000^[^10^]^. The annual percentage change (APC) and its 95% confidence interval (CI) in AAMRs were calculated using the Joinpoint Regression Program (Version 5.4, National Cancer Institute)^[^11^]^. This research systematically analyzes nationwide trends in mortality associated with the co-occurrence of AF/AFL and CKD in older adults (≥65 years) over the 26-year period from 1999 to 2024. This analysis fits log-linear models to detect points where mortality trends significantly changed in direction or magnitude. APCs were considered increasing or decreasing if the slope describing the change in mortality was significantly different from zero using two-tailed *t*-testing. Statistical significance was set at *P* < 0.05. Joinpoint regression analysis was used to evaluate the temporal trends in AAMRs due to AF/AFL -related heart failure (AF/AFL-HF) from 1999 to 2024. Significant changes in trends were identified using jointpoint software. Furthermore, jointpoint software is used to evaluate. *P*-values (Prob > |*t*|) and corresponding 95% CIs were calculated to evaluate each APC’s statistical significance in our manuscript. A *P*-value < 0.05 was considered statistically significant.

## Results

Among U.S. adults aged ≥65 years with CKD and AF/AFL, a total of 203 662 deaths occurred between 1999 and 2024 (Supplemental Digital Content Table S1, available at: http://links.lww.com/MS9/B126). Of these, 97 115 (47.7%) occurred among women and 106 547 (52.3%) among men, indicating a slight male predominance. In terms of place of death, the largest proportion occurred at medical facilities 88 108 (43.3%), followed by nursing home/long term care facility 48 989 (24.0 %), decedents’ homes 46 734 (22.9%), and hospices 46 233 (22.7%), as detailed in Supplemental Digital Content Table S2 available at: http://links.lww.com/MS9/B126.

### Overall

**Figure 1.**
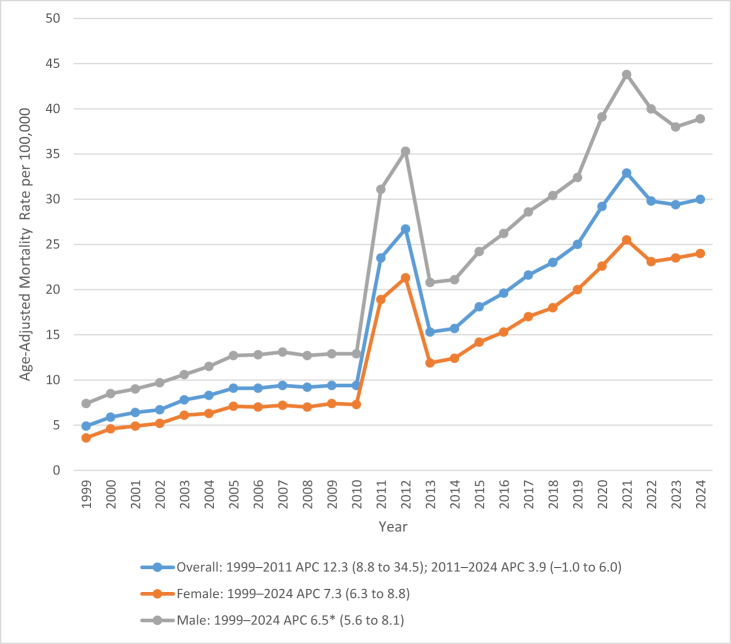
Age-adjusted mortality rates (AAMRs) for atrial fibrillation/flutter and chronic kidney disease-related deaths in the United States from 1999 to 2024, stratified by sex/gender.

Between 1999 and 2024, the overall AAMR for deaths due to AF and CKD increased markedly from 4.9 deaths per 100 000 population (95% CI: 4.7–5.2) in 1999 to 30.0 deaths per 100 000 (95% CI: 29.6–30.5) in 2024. From 1999 to 2011, AAMR increased from 4.9 (95% CI: 4.7–5.2) to 23.5 (95% CI: 23.1–24), signifying an evident increase during this period (APC: 12.28, 95% CI: 8.7–34.5, *P* = 0.0016). Then, from 2011 to 2024, mortality rose at a fluctuating rate reaching up to 30 (95% CI: 29.6–30.5) with an APC of 3.85 (95% CI: −1.02–5.9, *P* = 0.0767), as illustrated in Figure 1 and detailed in Supplemental Digital Content Tables S3 and S4, available at: http://links.lww.com/MS9/B126.

### Gender

Between 1999 and 2024, the AAMR for deaths related to AF and CKD was consistently higher among men (22.5 per 100 000; 95% CI: 21.8–23.2) compared with women (13.1 per 100 000; 95% CI: 12.7–13.5).

For women, there was a gradual increase in mortality with AAMR of 3.6 (95% CI: 3.4–3.9) in 1999 to 24 (95% CI: 23.4–24.5) in 2024 (APC: 7.3 95% CI: 7.32–6.28, *P* < 0.000001).

For men, there was a similar progression in mortality with AAMR of 7.4 (95% CI: 6.9–7.9) in 1999 to 38.9 (95% CI: 38.1–39.7) in 2024. (APC: 6.5 95% CI: 5.56–8.10 *P* < 0.000001).

Figure 1 illustrates these gender-specific differences, with corresponding data presented in Supplemental Digital Content Tables S3 and S4, available at: http://links.Lww.Com/MS9/B126.

### Race and ethnicity

Between 1999 and 2024, AAMRs for deaths related to AF and CKD varied markedly by race and ethnicity. NH White individuals had the highest AAMRs: 17.8 (95% CI: 17.4–18.2); followed by NH Black or African American: 16.4 (95% CI: 15.1–17.7); NH American Indian or Alaska Native: 15.3 (95% CI: 10.3–21.2); NH Asian or Pacific Islander groups: 9.7 (95% CI: 8.2–11.3); and Hispanic or Latino: 9.6 (95% CI: 8.5–10.8).

In NH White, Mortality increased steadily throughout the study period. From 1999 to 2024, AAMR rose from 4.9 (95% CI: 4.6–5.1) to 33.2 (95% CI: 32.5–33.6), corresponding to a significant APC of **+** 7.8 (95% CI: 6.9–9.1, *P* < 0.000001).

In NH Black or African American, AAMRs rose from 6.3 (95% CI: 5.3–7.3) in 1999 to 19.1 (95% CI: 17.5–20.6) in 2024, reflecting a consistent and significant increase over time (APC: +5.8, 95% CI: 4.8–7.4, *P* < 0.0227).

In NH American Indian or Alaska Native and NH Asian or Pacific Islander populations, mortality trends exhibited larger fluctuations, particularly in early years, likely reflecting small sample sizes and wide CIs. Among NH American Indian or Alaska Native, AAMRs increased from 11.5 (95% CI: 5.5–17.5) in 2004 to 23.9 (95% CI: 18.7–30.1) in 2022 (APC: +5.5%, 95% CI: −0.2–87.8, *P* = 0.0511), followed by a decline to 16.5 (95% CI: 12.3–21.7) in 2024 (APC: −20.8%, 95% CI: −45.1–7.3, *P* = 0.2639).

For NH Asian or Pacific Islander, rates rose modestly from 5.4 (95% CI: 3.8–7.3) in 1999 to 6.0 (95% CI: 4.7–7.6) in 2008 (APC: +1.5%, 95% CI: −23.2–12.5, *P* = 0.9866), increased sharply from 2008 to 2011 (APC: +42.9%, 95% CI: −3.6–64.7, *P* = 0.0523), declined between 2011 and 2014 (APC: −18.9%, 95% CI: −25.6–3.6, *P* = 0.0575), and rose again from 2014 to 2024 (APC: +5.5%, 95% CI: 2.3–12.1, *P* = 0.0228), reaching 14.9 (95% CI: 13.5–16.3) in 2024.

**Figure 2.**
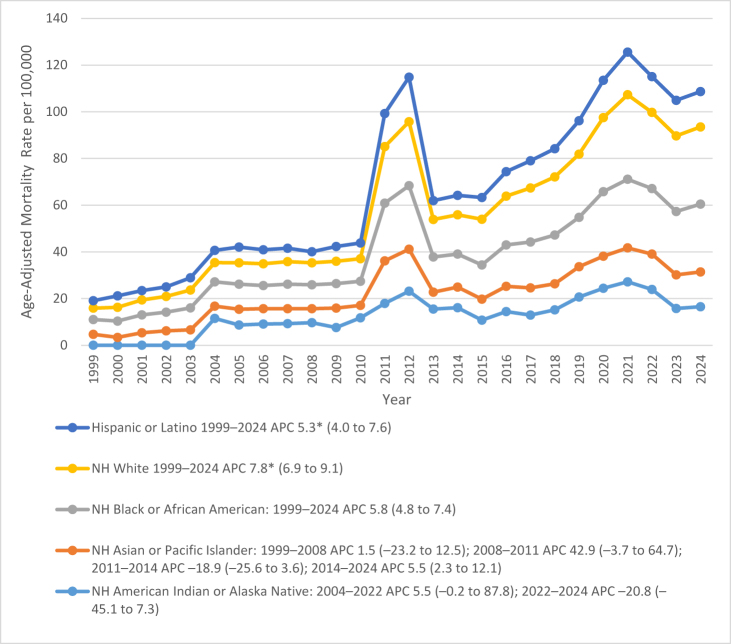
Age-adjusted mortality rates (AAMRs) for atrial fibrillation/flutter and chronic kidney disease-related deaths in the United States from 1999 to 2024, stratified by race/ethnicity.

In Hispanic or Latino, AAMRs increased significantly from 3.2 (95% CI: 2.3–4.4) in 1999 to 15.2 (95% CI: 14.1–16.3) in 2024, corresponding to an overall APC of +5.3 (95% CI: 4.0–7.6, *P* < 0.000001). Figure 2 illustrates these racial and ethnic differences, with corresponding data presented in Supplemental Digital Content Tables S3 and S5, available at: http://links.lww.com/MS9/B126.

### Census region

Between 1999 and 2024, AAMRs for deaths due to AF and CKD among older adults varied substantially by U.S. Census region. On average, over the course of the study period the highest mortality was observed in the Midwest region 19.0 (95% CI: 18.2–19.8), followed by the West 17.9 (95% CI: 17.1–18.7) and Northeast 15.6 (95% CI: 14.8–16.4), and lastly South 15.2 (95% CI: 14.7–15.8). Region-specific temporal patterns are summarized below.

In the Midwest, mortality rates rose sharply from 5.2 (95% CI: 4.7–5.7) in 1999 to 35.1 (95% CI: 34.1–36.2) in 2024, representing the highest overall regional rates by the study’s end. The APC of +7.5 (95% CI: 6.5–9.1, *P* < 0.000001) reflected the most rapid increase among all regions, with a marked acceleration after 2010.

In West, the AAMR increased from 4.7 (95% CI: 4.2–5.2) in 1999 to 31.8 (95% CI: 30.6–32.9) in 2012 with an APC of +13.3 (95% CI: 9.1–41.2, *P* = 0.0067), followed by a slower, non-significant rise to 31.2 (95% CI: 30.2–32.2) in 2024 with an APC of +3.9 (95% CI: −3.8 to 6.7, *P* = 0.1535).

In the Northeast, the AAMRs increased steadily from 5.1 (95% CI: 4.6–5.6) in 1999 to 26.1 (95% CI: 25.1–27.1) in 2024, highlighting a substantial APC of +6.6 (95% CI: 5.6–7.9, *P* < 0.000001).

**Figure 3.**
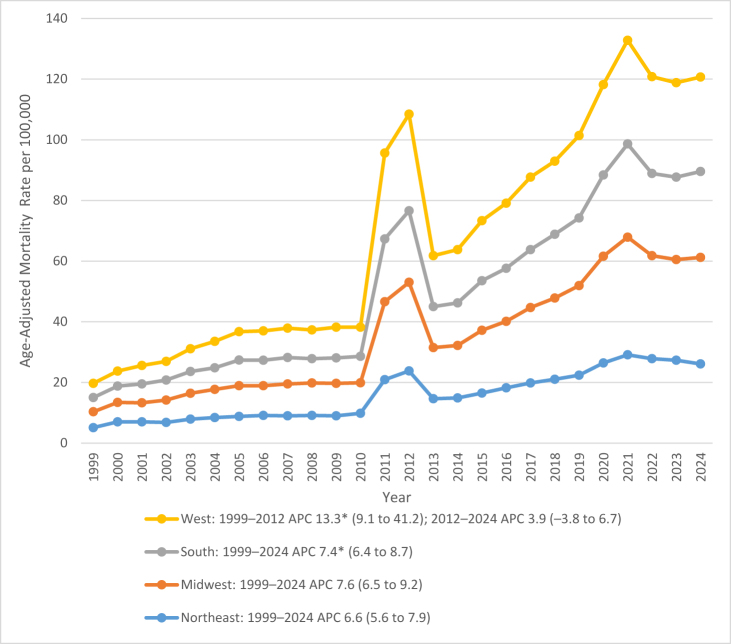
Age-adjusted mortality rates (AAMRs) for atrial fibrillation/flutter and chronic kidney disease-related deaths in the United States from 1999 to 2024, stratified by U.S. Census region.

In the South, the AAMRs increased gradually from 4.7 (95% CI: 4.3–5.1) in 1999 to 28.3 (95% CI: 27.6–29.1) in 2024 with an APC of +7.4 (95% CI: 6.4–8.7, *P* < 0.000001), though absolute rates remained lower than in the Midwest and West. Figure 3 illustrates these regional differences, with corresponding data presented in Supplemental Digital Content Tables S3 and S6, available at: http://links.lww.com/MS9/B126.

### Urbanization

Between 1999 and 2024, AAMRs for deaths due to CKD with AF/AFL and flutter increased markedly across both metropolitan and nonmetropolitan areas of the United States. Across the full period, the mean AAMR was higher in nonmetropolitan regions (18.9 per 100 000; 95% CI: 18.0–19.8) compared with metropolitan areas (16.3 per 100 000; 95% CI: 15.9–16.7). Mortality rose consistently in both populations, but the gap between them widened over time, particularly after 2010, when rates in nonmetropolitan areas accelerated more sharply.

In metropolitan areas, AAMRs rose progressively from 4.8 (95% CI: 4.5–5.0) in 1999 to 29.1 (95% CI: 28.6–29.6) in 2024. The increase was characterized by a rapid rise during the early 2000s, followed by a moderate but steady upward trend after 2012. During 1999–2012, mortality increased significantly (APC: +10.4, 95% CI: 7.5–34.3, *P* = 0.0151), with a slower but continued rise from 2012 to 2024 (APC: +4.7, 95% CI: −4.55 to 7.08, *P* = 0.1475). Despite some deceleration, overall mortality continued to climb throughout the study period.

**Figure 4.**
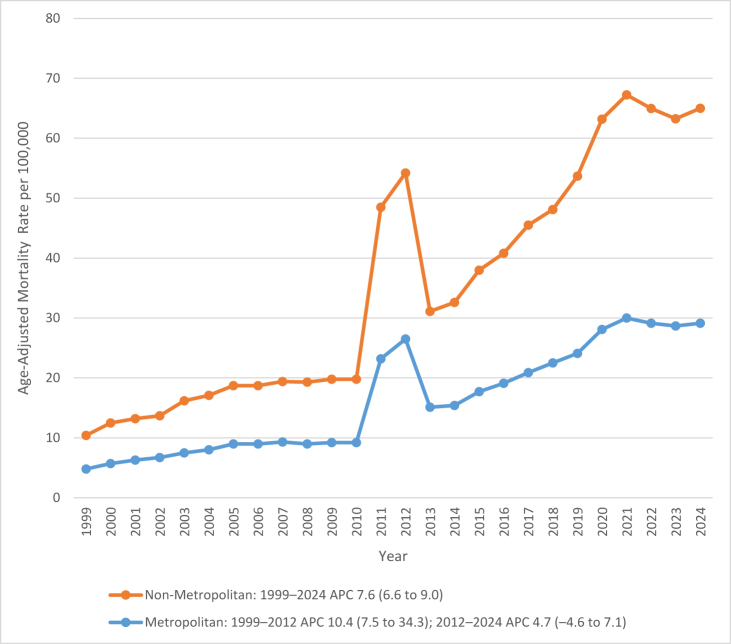
Age-adjusted mortality rates (AAMRs) for atrial fibrillation/flutter and chronic kidney disease-related deaths in the United States from 1999 to 2024, stratified by urban–rural classification.

In nonmetropolitan areas, mortality remained higher than in metropolitan regions across all years, increasing from 5.6 (95% CI: 5.0–6.2) in 1999 to 35.9 (95% CI: 34.6–37.1) in 2024. The overall upward trend was strong and statistically significant (APC: +7.6, 95% CI: 6.6–9.03, *P* < 0.0000010), indicating a consistent long-term rise without notable periods of decline. The rate of increase was particularly evident after 2010, resulting in substantially higher mortality among nonmetropolitan populations by 2024. Figure 4 illustrates these urbanization-level differences, with corresponding data presented in Supplemental Digital Content Tables S3 and S7, available at: http://links.lww.com/MS9/B126.

### State

**Figure 5.**
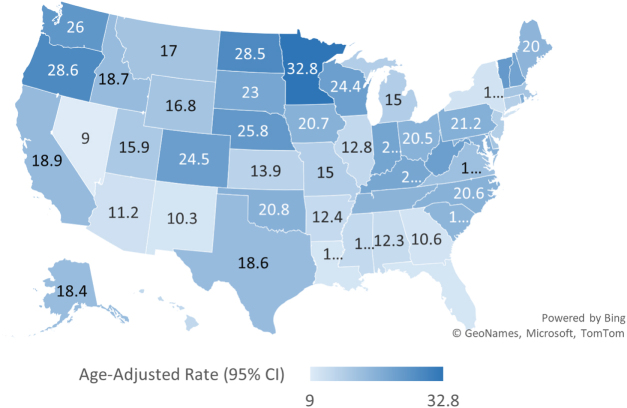
Age-adjusted mortality rates (AAMRs) for atrial fibrillation/flutter and chronic kidney disease-related deaths in the United States from 1999 to 2024, stratified by state.

State-level analysis revealed considerable variation in mortality burden associated with AF and CKD across the United States. The highest AAMRs were observed in Minnesota (32.8, 95% CI: 31.5–33.7), Oregan (28.6, 95% CI: 27.9–30.8), North Dakota (28.5, 95% CI: 25.8–30.1), Nebraska (25.8, 95% CI: 23.5–27), and Vermont (25.5, 95% CI: 22.8–28.2). In contrast, the lowest AAMRs were reported in Nevada (9.0, 95% CI: 8.1–9.9), Florida (10.3, 95% CI: 10.4–10.8), Louisiana (10.3, 95% CI: 9.7–11.2), New Mexico (10.3, 95% CI: 9.4–11.4), and Georgia (10.6, 95% CI: 10.1–11.1). Figure 5 illustrates these state-level differences, with corresponding data presented in Supplemental Digital Content Table S8, available at: http://links.lww.com/MS9/B126.

## Discussion

**Figure 6.**
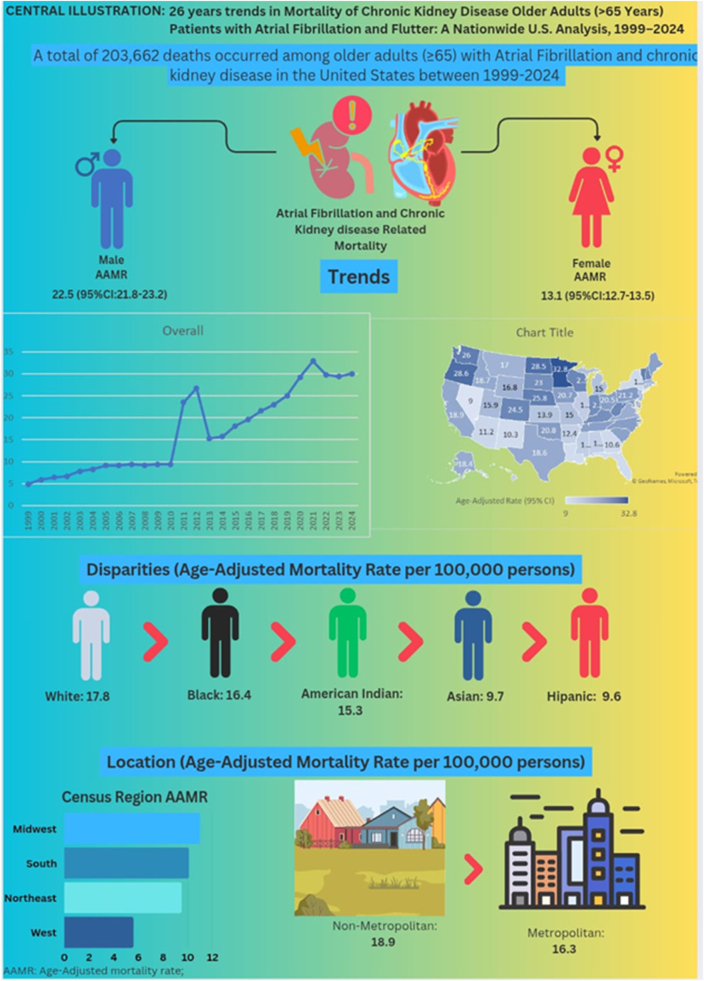
Demographic characteristics of atrial fibrillation/flutter and chronic kidney disease-related mortality in the United States from 1999 to 2024**/**central illustration.

The mortality rate among older persons (>65 years) in the United States who have AF/AFL and concurrent CKD increased significantly between 1999 and 2024, according to this national analysis. AAMRs increased steadily for both sexes, all racial and ethnic groupings, and all census regions during the research period, with overall deaths more than quintupling. Black or African American and NH White populations had the most marked increases in mortality, while men were consistently higher than women. The Midwest and West had the highest mortality rates, whereas the South and Northeast had comparable increase trends. Geographical inequalities were also apparent. Notably, the increase was more in non-metropolitan counties than in metropolitan ones, indicating growing disparities between rural and urban areas. Regional variation was further emphasized by state-level studies, which showed that Florida and Nevada had some of the lowest mortality rates, and Minnesota, Oregon, and North Dakota had the highest. All of these results point to a consistent and unequal rise in AF-CKD-related mortality among older persons in the United States, highlighting the critical need for focused clinical and public health initiatives to alleviate this dual illness burden (Fig. 6).

Individuals with CKD are at heightened risk for cardiovascular complications, including heart failure, coronary heart disease, and death, as well as AF^[^12^]^. Coronary artery disease, diabetes, and hypertension are among the risk factors that AF and CKD have in common, making them closely related illnesses^[^13^]^. Oxidative stress, systemic inflammation, and volume overload are brought on by CKD’s activation of the sympathetic nervous system and the renin-angiotensin-aldosterone system^[^14^]^. Atria structural and electrical remodeling is linked to angiotensin II’s ability to raise atrial pressure, hasten atrial fibrosis, and induce ion channel malfunction, all of which can lead to AF. These elements have a strong correlation with the structural and electrical remodeling of the atrium, which aids in the onset of AF.

We observed a sharp increase in mortality after 2010, building on a trend of rising deaths since 1999. This pattern may reflect limited access to healthcare prior to the implementation of the Affordable Care Act and potential downstream effects of the 2008 financial crisis^[^15^]^. Similar early-2010s surges have been noted in studies of patients with acute myocardial infarction and underlying CKD, followed by modest reductions in mortality potentially related to improvements in diagnosis, timely referrals, and evidence-based treatments^[^16^]^. Early therapies and public health initiatives may also have contributed to declines in AAMRs in certain CKD populations.

In our study, however, mortality among adults with concurrent AF and CKD continued to rise, which may be associated with an aging population and an increasing burden of cardiovascular comorbidities, including AF and declining renal function. By 2050, adults aged ≥65 years are projected to comprise up to 20% of the U.S. population^[^17^]^, suggesting that age-related risk factors may partially contribute to observed trends. Comparisons with studies examining cardiovascular disease as the underlying cause of death in patients with AF and CKD suggest that additional factors such as diabetes, obesity, and hypertension might play a role in elevated mortality^[^18,19^]^. Overall, the sustained increase in mortality among individuals with coexisting AF and CKD highlights a potentially vulnerable subgroup, warranting further research to better understand contributing factors, optimize management strategies, and reduce preventable deaths.

The observed gender gap can be partially explained by the fact that men’s CKD progresses more quickly^[^20^]^, and they are more likely to experience cardiovascular events^[^21^]^. One of the main reasons why males with CKD are more likely to experience negative consequences than women may be that they have greater systolic blood pressure^[^22^]^. The discrepancy shown in our study is also exacerbated by gender variations in CKD detection, recognition, monitoring, referrals, and management^[^23^]^. In contrast to women, men may have a faster loss in kidney function due to the protective impact of estrogen, the negative effects of testosterone, and hazardous lifestyle choices that are frequently observed in men^[^24^]^. According to our data, men are more likely to die from risk factors such as obesity, smoking, diabetes, and hypertension^[^25^]^, which may also be the cause of their higher death rates. These biological and socio-behavioral variations probably work together to explain the increased death burden seen in this analysis among men with AF and CKD.

NH Whites had a greater death rate than NH Blacks, according to our data. This might be at odds with recent research that indicates NH Blacks are more likely to die from AF^[^26^]^ and CKD^[^27^]^. The high prevalence of AF among NH White patients, which is more than double that of all other racial groups and accounts for 8% of the overall White population, may be the reason for our data’s suggestion of greater mortality among NH Whites^[^28^]^. Interestingly, while being more exposed to linked risk factors, our data also showed that Hispanics had the lowest AAMR. This has been referred to as the “Hispanic paradox” in the literature^[^29^]^. Regarding genetic susceptibility, Whites are more prone to AF compared to Blacks, as one of the single-nucleotide polymorphisms (SNPs) with an established link with AF, rs10824026, may partially mediate the greater risk of arrhythmia among Whites compared with Blacks. Black people are much more likely than White people to carry the minor allele of this SNP, which is known to be protective against AF^[^30^]^. On the other hand, the existence of particular APOL1 gene variants is a significant genetic risk factor for CKD, especially among Black Americans, as they are associated with an increased risk of renal disease and a quicker progression to kidney failure. The incidence of ESRD is significantly higher in Black people than in White people^[^31^]^. We saw a decline in mortality among American Indians after 2022, but the literature indicates that this might not be a true improvement in health but rather could be a result of changes in population demography, health reporting, or data collection techniques. A high incidence of risk factors, the disproportionate effect of COVID-19 on this group, and racial misrepresentation in mortality statistics are likely contributing causes to the observation. All things considered, these racial and ethnic disparities most likely result from a combination of demographic, medical, and genetic factors. Similar to Hispanics, Asian, and Pacific Islander communities had lower death rates, which may be related to healthier lifestyles and better preventative care. In contrast, NH Whites may have higher rates due to genetic predisposition and a higher prevalence of AF.

Although our findings show the highest mortality rates due to AF and CKD in the western region, regional mortality distribution has been noted to be greatest in the South, followed by the West, Midwest, and North^[^16,32,33^]^. This may be due to population aging and comorbidity burden. The Midwest and West have higher proportions of older adults with multiple comorbidities, which amplify AF–CKD mortality risk^[^34,35^]^. Regional differences in diet, physical activity, and smoking prevalence may also influence cardiovascular and renal outcomes. For example, higher obesity and diabetes rates in certain Midwestern counties can worsen CKD progression and AF complications^[^36^]^.

People living in rural areas were more likely to die from CKD than people living in urban areas, possibly because urban areas tend to have more tertiary care hospitals, which would allow for more extensive CKD screening programs. Transportation barriers and barriers to dialysis care at home or in a facility affect treatment decisions for some patients^[^37,38^]^. Socioeconomic factors, such as lower income, low health literacy, and higher rates of uninsured people, also lead to poor management of chronic conditions such as diabetes and hypertension, which are major risk factors for CKD^[^39^]^, and higher rates of smoking, obesity, and lack of access to healthy foods may also worsen CKD mortality in these areas^[^40,41^]^. Overall, these regional and urban–rural disparities underscore the influence of sociodemographic, lifestyle, and healthcare access factors on AF and CKD mortality, highlighting the need for region-specific prevention strategies and improved access to specialized cardiovascular and renal care.

The necessity for coordinated multidisciplinary methods for prevention and care is highlighted by the increasing mortality burden among older persons who have both CKD and AF. Finding high-risk categories, enhancing early diagnosis, and resolving inequities in healthcare access and quality should be the top priorities of future study. To counteract these tendencies, it will be crucial to promote the equitable use of evidence-based treatments and to expand national surveillance systems.

### Limitations

It is important to recognize several limitations of this study. First, the analysis relies on mortality data from death certificates, which may be misclassified or underreported, particularly for race and comorbid conditions such as AF and CKD. Second, the study is descriptive and observational, limiting the ability to draw causal inferences regarding the relationship between mortality trends and the coexistence of AF and CKD. Third, although the study provides national-level insights, observed differences may be influenced by the absence of individual-level data on clinical management, socioeconomic factors, and treatment adherence. Mortality estimates may also have been affected by changes in diagnostic criteria, cause-of-death coding practices, and access to healthcare over the 26-year study period. Finally, the study population was restricted to adults aged ≥65 years, which may limit generalizability to younger individuals with early onset CKD or AF.

## Conclusion

In summary, the persistent disparities in disease burden and healthcare outcomes among older adults in the U.S. are underscored by the ongoing rise in AF–CKD-related mortality across demographic and regional groups. For patients affected by this complex comorbidity, these findings advocate for immediate, concerted efforts that integrate clinical optimization, population-based prevention, and health policy initiatives to enhance survival, reduce inequities, and improve care quality.

## Data Availability

The dataset supporting the conclusions of this article is included in this article.
